# Myostatin and other musculoskeletal markers in lung transplant recipients

**DOI:** 10.1007/s10238-018-0532-3

**Published:** 2018-10-13

**Authors:** Katharina Kerschan-Schindl, Gerold Ebenbichler, Wolfgang Gruther, Ursula Föger-Samwald, Stefan Kudlacek, Janina Patsch, Andreas Gleiss, Peter Jaksch, Walter Klepetko, Peter Pietschmann

**Affiliations:** 1 Department of Physical Medicine, Rehabilitation and Occupational Therapy, Medical University of Vienna, Vienna, Austria; 2 Department of Pathophysiology and Allergy Research, Center of Pathophysiology, Infectiology, and Immunology, Medical University of Vienna, Vienna, Austria; 3 Barmherzige Brueder Hospital, Vienna, Austria; 4 Department of Biomedical Imaging and Image-guided Therapy, Medical University of Vienna, Vienna, Austria; 5 Center of Medical Statistics, Informatics, and Intelligent Systems, Medical University of Vienna, Vienna, Austria; 6 Department of Surgery, Medical University of Vienna, Vienna, Austria; 7 Present Address: healthPi, Vienna, Austria

**Keywords:** Lung transplantation, Myostatin, Follistatin, Sclerostin, Dickkopf 1, Periostin

## Abstract

Recipients of lung transplantation (LuTx) may experience impaired muscle function and bone metabolism even after rehabilitation. We investigated the potential use of musculoskeletal markers in identifying the impairment of muscle function and bone function in these patients. Biochemical parameters, bodily functions, and lung function of 37 LuTx recipients were evaluated at the time of their discharge from the hospital stay and about 6 months later. The biomarkers were also assessed in 30 healthy age and gender distribution-matched controls. Compared to controls, the negative muscle regulator myostatin was elevated in LuTx recipients at baseline and follow-up, whereas its opponent follistatin only showed a group-specific difference at follow-up. LuTx recipients had reduced serum levels of sclerostin and increased levels of dickkopf 1 and periostin. Lung function and physical function were improved during follow-up. The change in lung function was correlated with the change in chair-rising time and the 6-min walking test. At follow-up, all musculoskeletal markers of LuTx recipients differed from those of controls, thus reflecting their still reduced lung function and bodily functions. Among the tested biomarkers, myostatin, sclerostin, dickkopf 1, and periostin were useful to detect impaired musculoskeletal function in LuTx recipients. Myostatin may serve as a target of treatment in the future.

## Introduction

While the mean life expectancy of the general population has risen steadily throughout the world, the prevalence of patients suffering from a chronic progressive end-stage disease requiring organ transplantation has also increased [1]. The duration of solid organ allograft survival has been prolonged: the half-life of lung transplants was 1.7 years in 1989 and 5.2 years in 2005 [2]. Thus, currently transplant recipients are more likely to live to an advanced age. However, their health may be poorer than that of the general population. Thus, the search for diagnostic regimens including surrogate markers to identify predictors of frailty, sarcopenia, and osteoporosis, as well as effective therapy strategies for these conditions, is of great interest for these patients.

Chronic organ failure is correlated with comorbidities such as musculoskeletal disease, progressive muscle tissue wasting, and bone mineral loss. Physical inactivity, inflammation, oxidative stress, and corticosteroid use are all known to contribute to skeletal muscle weakness as well as osteoporosis. Reduced muscle function is caused by fiber atrophy as well as a fiber shift toward a glycolytic phenotype and, consequently, impaired oxidative capacity. More than 85% of patients with chronic obstructive pulmonary disease (COPD) who are eligible for pulmonary rehabilitation were found to be sarcopenic [3]. Their reduced quadriceps strength served as a useful predictor of mortality [4]. Bone metabolism disorders are a common condition in patients with solid organ failure. Especially those with end-stage lung disease as well as lung transplant (LuTx) recipients appear to be severely affected by bone metabolism disorders. Compared to patients with end-stage liver, kidney, or heart failure awaiting transplantation, the highest prevalence of osteoporosis (67%) has been noted in patients with end-stage lung disease [5].

LuTx is frequently the only therapy option for patients with end-stage lung disease. Although their quality of life usually improves very markedly after transplantation, their function may remain inferior to that of healthy subjects [6, 7]. LuTx recipients clearly have a lower level of endurance than healthy age-matched controls [8]. Their pre-transplant reduction in muscle mass and quadriceps strength was found to persist for up to 3 years after surgery [9]. Thus, despite increasing survival rates after LuTx, clinicians need to focus not only on early complications such as primary graft dysfunction or acute rejection, but also on complications such as myopathy or osteoporosis because bone loss continues to persist after transplantation. This holds true for all solid organ transplantations, but the highest risk of osteoporosis and fracture was noted in LuTx recipients [10].

The evaluation of muscle and bone status is an essential aspect of a patient’s rehabilitation after LuTx. Besides imaging techniques and functional assessment, biochemical parameters may provide valuable diagnostic information about musculoskeletal health and permit basic science as well as clinical care to jointly address the common goal of alleviating the devastating impact of sarcopenia and osteoporosis. Normal muscle mass and function are believed to depend on the balance between positive regulators of muscle growth, including follistatin (FSTN), and negative regulators such as myostatin (MSTN) [11]. MSTN and FSTN are muscle markers used for research purposes. FSTN prevents MSTN from binding to its receptor and thus opposes its effect. Elevated serum levels of MSTN were noted in persons with age-associated sarcopenia and in patients with a variety of chronic diseases [12]. The uncoupling of bone formation and bone resorption leads to changes in bone mass. Sclerostin (SOST) and dickkopf-1 (Dkk 1) are inhibitors of the Wnt signaling pathway and thus reduce bone formation and regeneration [13]. A preferentially in periosteal tissue located biochemical parameter is periostin (PSTN), the osteoblast-specific factor 2, which is supposed to be a marker of periosteal metabolism contributing to bone strength [14]. None of these musculoskeletal biomarkers have been investigated in LuTx recipients; their relevance as blood-based measures of impaired muscle function and bone function remains unclear.

The aim of the present study was to evaluate differences in muscle and bone markers between LuTx recipients and healthy controls and, in patients, assess potential short-term changes in physical function, lung function, and biochemical parameters. A better understanding of changes in the musculoskeletal system after transplantation may help to improve the treatment of muscle and bone alterations in LuTx recipients.

## Patients and methods

### Study population

Unilateral or bilateral LuTx recipients were evaluated prospectively. The study was approved by the ethics committee of the Medical University of Vienna (IRB approval number 999/2011) and performed in accordance with the ethical standards of the 1964 Declaration of Helsinki and its subsequent amendments. All participants provided their written informed consent after the procedure of the trial had been explained to them. Participants had to be at least 18 years of age. A minimum capacity of physical mobility was required: standing without any device for at least 5 min and walking a distance of at least 50 meters with or without a walking aid. We enrolled 37 LuTx recipients during the first 2 days after their acute hospital stay. Pre-transplantation (before study enrollment) drug regimens were specific to the underlying lung disease and the respective patient’s needs and were not standardized. However, all patients received oral low-dose corticosteroids (5–10 mg daily). The immunosuppressive regimen after transplantation was standardized. It consisted of a calcineurin inhibitor (tacrolimus, 0.05–0.1 mg/kg^−1^/day; or cyclosporine A, 5–10 mg/kg^−1^/day), mycophenolate mofetil (2–3 g/day), and corticosteroids (methylprednisolone, 125 mg every 8 h for the first 24 h, followed by prednisolone 1 mg/kg^−1^/day). Gradually, prednisolone was reduced to 5 mg/day. During the first 90 days after transplantation, all patients received vancyclovir (450 mg twice a day) for the prevention of a cytomegalovirus infection. Bone-specific medication was initiated by the treating physician.

The rehabilitation program was started immediately after discharge from the acute hospital stay. The duration of the post-acute rehabilitation period was 1–2 months and was tailored to the individual patient’s needs. As already described [15], Austrian citizens received an inpatient rehabilitation, and foreign patients for economic reasons an outpatient rehabilitation program which was similar but less intensively supervised. It included endurance, strength, and strength endurance training with elastic bands for all major muscle groups. Whereas inpatient rehabilitation lasted for 4–6 weeks with supervised training intervention 4 times per week, the outpatient rehabilitation program lasted for 8–10 weeks with supervised sessions twice a week. These patients were strongly encouraged to walk as briskly as possible every day for up to an hour, and climb stairs. In addition, patients were taught to perform regulatory respiratory exercises to increase respiratory muscle strength and to improve/maintain optimal clearance of sputum (bronchial hygiene therapy). Depending on the decision of the attending physician, LuTx recipients were offered nutritional and psychological counseling.

Pulmonary function was evaluated at all checkups during the patients’ routine follow-up care. Forced expiratory volume in one second (FEV_1_), vital capacity (VC), and total lung capacity (TLC) were assessed.

### Biochemistry

Regular biochemical analyses were performed during the acute hospital stay; these included the measurement of serum calcium, phosphate, creatinine, protein, alkaline phosphatase, gamma-glutamyltransferase, and 25-OH-vitamin D. All analyses were conducted according to standard procedures.

Venous blood samples were taken twice: at the time of hospital discharge and about 6 months later. Samples were collected in the morning to eliminate diurnal variations in the biochemical variables. Serum was separated from whole blood by centrifugation and then immediately frozen and stored at − 70° until assayed. All samples were handled in a single batch run. The following musculoskeletal markers were investigated: *myostatin* (MSTN, colorimetric competitive immunoassay, Immundiagnostik, Bensheim, Germany, limit of blank LoB 0.370 ng/ml; intra-assay coefficient of variation < 12%, inter-assay coefficient of variation < 14%, according to the manufacturer’s data), *follistatin* (FSTN, colorimetric sandwich immunoassay, R&D Systems, Minneapolis, USA, MDD range 0.005–0.068 ng/mL; mean MDD 0.016 ng/mL; intra-assay coefficient of variation < 3%, inter-assay coefficient of variation < 10%, according to the manufacturer’s data), *sclerostin* (SOST, BI-20492, colorimetric sandwich immunoassays, Biomedica, Vienna, Austria; detection limit 3.2 pmol/l; intra-assay coefficient of variation ≤ 7%, inter-assay coefficient of variation ≤ 10%, according to the manufacturer’s data), *dickkopf 1* (Dkk 1; BI-20412, colorimetric sandwich immunoassays, Biomedica, Vienna, Austria; detection limit 0.38 pmol/l; intra-assay coefficient of variation ≤ 8.0%, inter-assay coefficient of variation ≤ 12.0%, according to the manufacturer’s data), and *periostin* (PSTN; SEH339Hu, colorimetric sandwich immunoassays, Cloud-Clone-Corp, Houston, USA; detection limit 0.068 ng/ml; intra-assay coefficient of variation ≤ 10%, inter-assay coefficient of variation ≤ 12%, according to the manufacturer’s data).

Additionally, serum levels of these biomarkers were evaluated in a healthy age-matched control group; these 30 subjects were part of a previously published population-based cohort [16].

### Functional assessment

Performance-based physical function was assessed at baseline and about 6 months later. The measures were strength, mobility, and exercise capacity tests. Leg power was measured by the time it took the patients to stand up and sit down five times as quickly as possible from a chair of standard height (46 cm seat height) with their arms fold across their chests [17]. The timed up and go test (TUG) [18] assesses mobility and measures, in seconds, the time it takes for an individual to stand up from a chair, walk a distance of 3 meters, turn around, walk back to the chair, and sit down again. The 6-min walking test (6MWT) is a proxy of functional capacity and was performed according to the American Thoracic Society guidelines [19]. Patients walked as quickly as they could on a level course for six minutes, and their walking distance was evaluated. Maximal isometric grip strength was assessed with a Jamar dynamometer (Asimow Engineering Co., Los Angeles, CA, USA). Measurements were obtained in sitting position, with patient’s shoulders adducted to the body, the elbows flexed to 90°, and the forearms and wrists in neutral position. Patients were instructed to press three times as hard as possible on each side [20]. The mean of the two better tests was used for the analysis.

### Bone mineral density measurement

Bone mineral density (BMD) was measured at the lumbar spine and the hip region in a subgroup of 17 patients at the department of biomedical imaging and image-guided therapy, Medical University of Vienna. A dual energy X-ray absorption device (DXA; HOLOGIC 4500; Hologic Inc, Waltham, MA, USA; CV 2%) was used. All measurements were performed according to the standard procedure recommended by the manufacturer.

### Statistical analysis

Categorical variables are described by counts and percentages. The distributions of continuous variables are presented as medians [quartiles] and box plots due to non-symmetric distributions and compared between independent groups using Wilcoxon’s rank-sum test. Wilcoxon’s signed-rank test was used to test null hypotheses of no intra-individual change. The association between intra-individual changes in parameters of lung function and physical function was quantified using Spearman’s correlation coefficients.

The two-sided significance level was set to 0.05. No correction for multiple testing was used because of the exploratory nature of the study. *P* values and confidence intervals must be interpreted accordingly. All calculations were performed with SAS 9.4 (SAS Institute Inc., Cary, NC, USA, 2012).

## Results

### Study participants

**Fig. 1:**
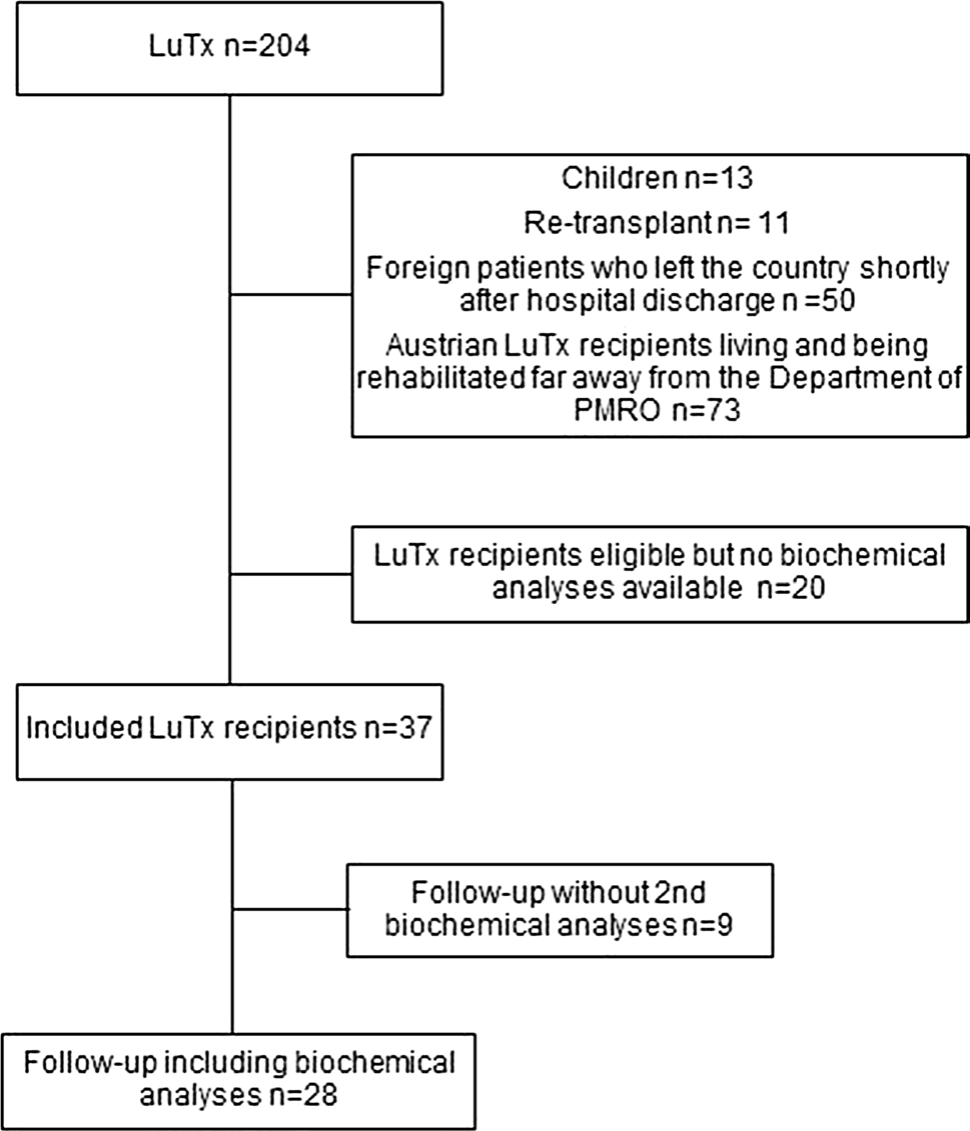
Flowchart of the study population

During the period of inclusion in the study, 204 patients underwent a LuTx. Fifty nonresident patients left the country shortly after they were discharged from the hospital. Seventy-three Austrian LuTx recipients lived at a distance from Vienna and were referred to another rehabilitation center. Thirty-seven LuTx recipients (23 men and 14 women)—foreign patients who underwent rehabilitation at our department as well as Austrian patients who underwent rehabilitation at a different center but were willing to report to our department for assessment—were eligible for functional assessment and biochemical evaluations at baseline (Fig. 1). Their median age was 53.0 [33.0; 58.0] years and their body mass index 22.0 [19.0; 25.4]. Indications for transplantation included chronic obstructive pulmonary disease (COPD; *n* = 14; 37.8%), interstitial lung disease (*n* = 11; 29.7%), cystic fibrosis (*n* = 7; 18.9%), and other etiologies (*n* = 5; 13.5%) including pulmonary hypertension, alveolar microlithiasis, and congenital bronchiectasis. All but two patients had undergone bilateral LuTx. BMD measurement at baseline revealed osteopenic values with a T score of − 1.7 [− 2.5; − 1.1] at the lumbar spine, and − 1.9 [− 2.5; − 1.2] at the femoral neck. Eight patients regularly took a bisphosphonate (alendronate) plus calcium and vitamin D; 16 patients received supplementation with calcium and vitamin D only. Follow-up biochemical analyses were performed 6.5 [5.2; 7.6] months after baseline assessment and were available for 28 LuTx recipients (Fig. 1). The healthy control group (distribution matched according to age, gender, and BMI) consisted of 21 men and 16 women; their median age was 53.1 [29.9; 59.1] years.

### Biochemical parameters

**Fig. 2:**
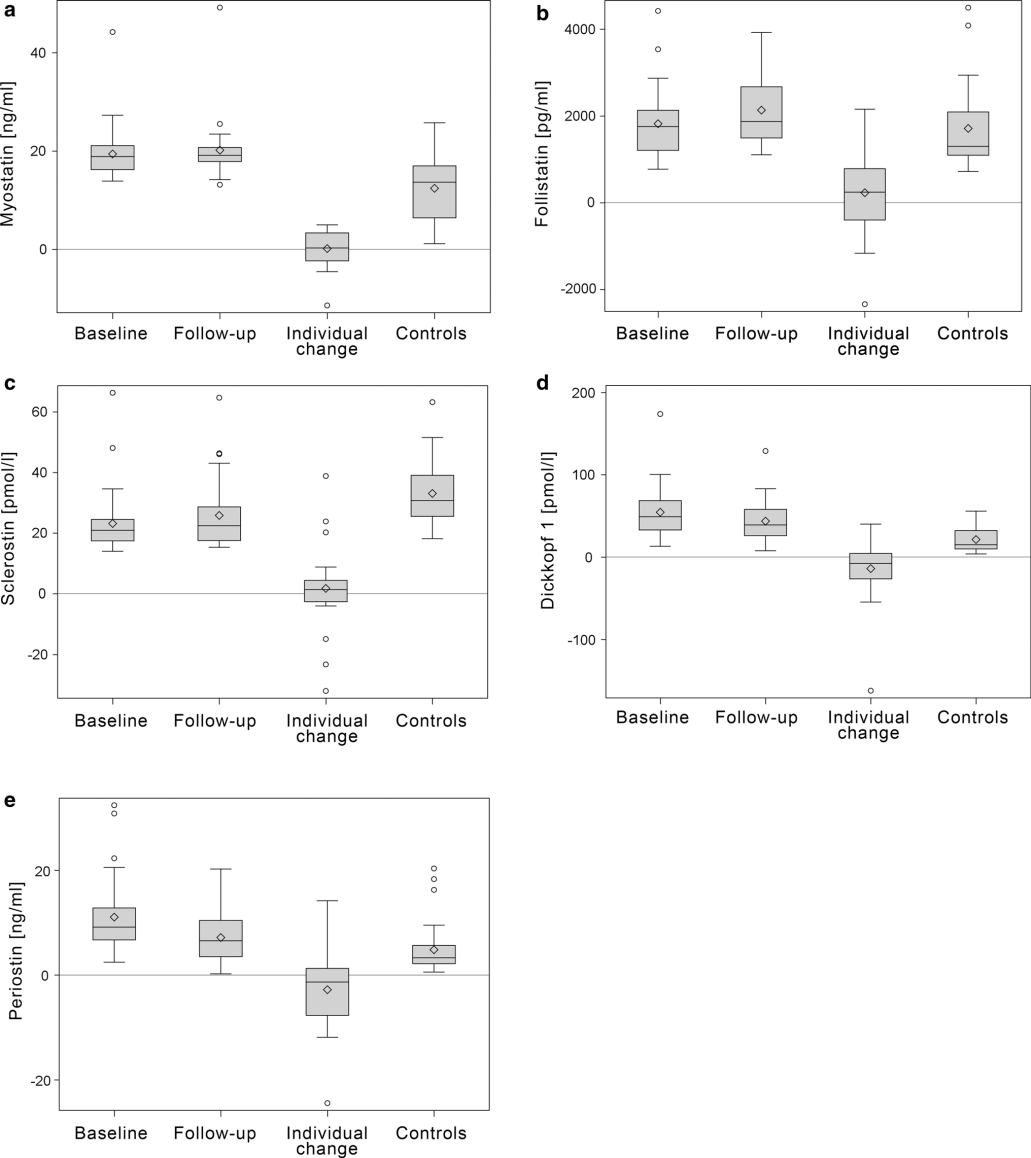
Musculoskeletal markers: baseline data and individual changes compared to healthy controls

At baseline, the patients’ median renal function was normal but their gamma-glutamyltransferase levels were high. Serum levels of vitamin D were well below the lower limit (Table 1). MSTN concentrations were significantly higher in LuTx recipients than in controls. No such group-specific differences were identified for FSTN. Group comparison also showed lower SOST but higher Dkk 1 and PSTN concentrations in LuTx recipients (Table 2). The changes in these biochemical parameters during follow-up were rather small and did not achieve statistical significance (Fig. 2).

**Table 1:** Baseline values of biochemical parameters for LuTx recipients (*n* = 37)

Biochemical parameter	Baseline	Reference range
Calcium (mmol/l)	2.25 [2.14; 2.34]	2.15–2.50
Phosphate (mmol/l)	0.77 [0.63; 0.94]	0.81–1.45
Creatinine (mg/dl)	0.71 [0.48; 0.97]	0.50–0.90
Protein (g/l)	63.6 [58.9; 68.2]	64–83
Alkaline phosphatase (U/l)	80.0 [49.5; 112.8]	35–105
GGT (U/l)	102.0 [55.3; 192.8]	< 40
25-OH-Vitamin D (mmol/l)	16.6 [10.9; 33.8]	75–250

*GGT* gamma-glutamyltransferase; median [quartiles]

**Table 2:** Musculoskeletal markers: LuTx recipients and controls at baseline

Biochemical parameter	LuTxR (*n* = 37)	Control group (*n* = 37)	*p*
MSTN (ng/ml)	18.87 [16.20; 21.08]	13.65 [6.40; 16.97]	< 0.001
FSTN (pg/ml)	1754.37 [1206.25; 2129.38]	1298.50 [1093.00; 2091.00]	0.244
SOST (pmol/l)	20.92 [17.43; 24.51]	30.74 [25.53; 39.05]	< 0.001
Dkk 1 (pmol/l)	48.97 [32.89; 68.53]	14.95 [9.83; 32.12]	< 0.001
PSTN (ng/ml)	9.16 [6.72; 12.83]	3.30 [2.18; 5.66]	< 0.001

*MSTN* myostatin, *FSTN* follistatin, *SOST* sclerostin, *Dkk 1* dickkopf 1, *PSTN* periostin

### Functional tests

At the time of discharge from the acute hospital stay, four LuTx recipients were unable to perform the chair stand test at least once. At follow-up, all subjects were able to perform the chair rise correctly. All tested variables of lung function and physical function were found to be significantly improved on the second day of assessment. However, some of these (6MWT and grip strength) were still clearly lower than published reference values for healthy controls of comparable age (Table 3).

**Table 3:** Lung function and physical function of LuTx recipients at baseline and follow-up

Outcome measure	Baseline	Follow-up	Individual change	*p*	Normal value
VC (%)	49.3 [39.5; 61.4]	77.9 [62.8; 87.0]	21.6 [14.8; 36.10]	< 0.001	100
FEV1s (%)	52.8 [43.9; 69.2]	84.0 [59.3; 94.7]	23.0 [8.7; 34.5]	< 0.001	100
TLC (%)	69.6 [64.2; 82.0]	83.3 [75.9; 101.1]	8.8 [5.2; 16.6]	< 0.001	100
Chair rise (s)	13.1 [11.5; 18.2]	10.0 [7.4; 11.1]	− 4.0 [− 8.6; − 2.4]	< 0.001	≤ 10 [17]
TUG (s)	8.4 [7.1; 9.2]	6.0 [5.1; 6.5]	− 1.9 [− 3.4; − 1.0]	< 0.001	< 10 [18]
6MWT (m)	378 [327; 449]	542 [470; 619]	140 [63; 225]	< 0.001	600 [21]
Grip strength left (kgF)	14.0 [9.5; 27.0]	28.0 [16.5; 36.5]	4.0 [2.5; 8.0]	0.0113	M 43, F 26 [22]
Grip strength right (kgF)	15.5 [10.5; 28.5]	27.0 [17.5; 37.5]	5.5 [1.5; 10.5]	0.0382	M 45, F 28 [22]

*VC* vital capacity, *FEV1s* forced expiratory volume in 1 s, *TLC* total lung capacity, *TUG* timed up and go test, *6MWT* 6-min walking test; median [quartiles]; *M* males, *F* females

### Relationship between lung function and physical function

Correlation analyses between changes in lung function and physical function revealed a strong negative relationship between the change in chair rise and changes in all pulmonary function parameters. A moderate correlation was noted between the change in the 6MWT and changes in VC and FEV1s (Table 4).

**Table 4:** Correlation analyses (Spearman’s correlation coefficients): changes in lung function and physical function

	∆VC (%)	∆FEV1s (%)	∆TLC (%)
∆Chair rise (s)	− 0.717*	− 0.780*	− 0.601*
∆TUG (s)	− 0.378	− 0.300	− 0.251
∆6MWT (s)	0.562*	0.465*	0.400

∆6MWT, change in 6-min walking test; ∆TUG, change in timed up and go test; ∆VC, change in vital capacity; ∆FEV1s, change in forced expiratory volume in 1 s; ∆TLC, change in total lung capacity

*p < 0.05

## Discussion

To our knowledge, the present study is the first to evaluate serum myostatin levels in solid organ transplant recipients. We assessed further musculoskeletal markers as potential diagnostic or therapy targets in muscle and bone disease among LuTx recipients. As such, MSTN—a negative regulator of muscle growth—was significantly higher in LuTx recipients than in controls at both time points, whereas serum FSTN levels showed a significant difference only during follow-up. With regard to the negative regulators of bone mass, SOST was lower, whereas Dkk 1 and PSTN were higher in LuTx recipients immediately as well as 6 months after transplantation.

The elevated serum MSTN levels observed immediately after transplantation and also about 6 months later are in line with the data reported from previous investigations, showing increased levels of MSTN in cachectic states associated with a variety of health conditions [12] including COPD [23]. The increased expression of MSTN in skeletal muscle noted in three patients after liver transplantation [24] appears to corroborate the results of the present investigation. Higher MSTN levels in solid organ transplant recipients may at least be partly due to the administration of glucocorticoids and CsA [25, 26]. When discharged from the acute hospital stay after transplantation, LuTx recipients are typically restricted in their activities of daily living. The loss of regular physical activities is known to be related to the loss of muscle mass, which would fit well with the elevated serum levels of MSTN observed in transplant recipients compared to controls. However, this variable was not sensitive enough to detect the positive effect of rehabilitation on the state of muscles despite clinically relevant improvements in physical function tests. Interestingly, FSTN—the antagonist of MSTN—did neither differ from controls at baseline nor reveal significant changes thereafter. However, these nonsignificant increases in FSTN led to significantly higher FSTN levels compared to controls during follow-up. The increased levels of FSTN with increasing mobility and muscle function during the follow-up period were not very pronounced. The differences were nonsignificant and in line with two recent studies, showing that FSTN increased with exercise [27, 28]. However, in one of the studies the exercise was highly strenuous [28]. FSTN is a liver-derived molecule regulated by the glucagon-to-insulin ratio. Since conditions other than exercise are also liable to influence this ratio [29], FSTN appears to lack sensitivity in the detection of impaired muscle function. CsA was found to inhibit the expression of FSTN [30] which may, in part, explain the lack of a significant increase in FSTN with improved physical function.

SOST, a negative regulator of bone formation, is known to inhibit the Wnt/ß-catenin signaling pathway. Serum levels of SOST were significantly lower in LuTx recipients than in controls at baseline as well as during follow-up. This may be at least partly due to the administration of glucocorticoids in patients with chronic lung disease or after transplantation. In fact, immediately after transplantation a patient needs high glucocorticoid doses to prevent graft rejection. Despite a stepwise reduction in the dose over time, glucocorticoids are known to compromise muscle and bone metabolism even when taken in low doses [31]. Glucocorticoids were found to promote the apoptosis of osteocytes [32]; the latter are known to express SOST. As LuTx recipients have a reduced bone mass [5], fewer osteocytes would be available to produce SOST, thus explaining the low SOST levels observed in the transplant recipients examined in the present study.

Dkk 1, the second negative regulator of bone formation, was elevated in LuTx recipients. This is in accordance with in vitro examinations [33] but not with an in vivo investigation performed in a different clinical setting [34]. However, Dkk 1 may not be as bone-specific as SOST because it is also expressed in other tissues such as the skin. Disregarding this fact, Dkk 1 serum protein appeared to be a sensitive indicator of inactivity-related impairment of bone metabolism in the present study. This is especially true in view of the still impaired physical performance levels of LuTx recipients, as evidenced by their lower than normal reference values for the 6MWT and the hand grip strength test [35, 36]. In a recent animal study, the authors observed higher protein levels of Dkk 1 in sedentary rather than regularly exercising rats [37]. Ultra-distance runners had decreased Dkk 1 levels after a Spartathlon competition [28].

Serum levels of PSTN, the third bone marker assessed in this study, were higher in transplant recipients than in controls. This appears to contradict our expectation of reduced PSTN levels because it is known to correlate with periosteal bone formation [14]. However, in the OFELY study [38] postmenopausal women had higher serum periostin levels and a correspondingly higher risk of fractures. Likewise, LuTx recipients also had a higher risk of fragility fractures [10].

In the present study, the functional performance of LuTx recipients improved to a clinically relevant extent during follow-up. All patients underwent post-acute rehabilitation and were subsequently asked to maintain an active lifestyle in order to achieve their optimum exercise capacity [39]. In fact, the improvement in lung function and physical performance tests, providing an estimate of overall body strength (hand grip strength), leg power of the thigh muscles (time taken for five repetitions of the chair rise), cardiopulmonary capability (6MWT), and the risk of falls (TUG), were comparable with the data reported from previous research [40, 41]. Despite these remarkable beneficial functional changes, the results of the 6MWT and the hand grip strength test were still clearly below the reference values published for age- and sex-matched healthy controls, suggesting that the regular intake of immunosuppressant medication may affect the full recovery of muscle and endurance function in LuTx recipients. This is supported by the data concerning musculoskeletal markers in the present study. With the exception of FSTN, these markers appeared to be sensitive in detecting impairments in muscle function and bone function. The fact that physical function was not entirely restored at follow-up may explain the significant differences in all musculoskeletal markers between LuTx recipients and controls at this time point. Thus, these surrogate markers basically have the potential to reflect basic muscle and bone metabolic disorders, but will probably be unable to reflect the severity of impaired muscle function in immunosuppressed patients.

Lung function improved significantly after surgery and was associated with changes in physical function as well. The strong negative correlation between all parameters of pulmonary function and changes in the chair raising test imply that LuTx recipients experience better pulmonary function after surgery, which enhances their level of physical activity and the function of their thigh muscles. The impressive increase in functional capacity within the first few months after transplantation, assessed by the distance covered (140 [63; 225] m) in 6 min of walking, was significantly associated with ∆VC and ∆FEV1s.

One limitation of the present study is the fact that we had no data concerning the pre-transplant levels of blood markers for our patients. The reasons were that the actual time of surgery is unpredictable for patients listed to undergo LuTx and that one half of the patients who participated in the study were residents of foreign countries. A one-to-one matched control group would be more accurate than the distribution-matched control group used in the study, but would probably not have altered the results. Unfortunately, we are not able to discriminate between the impact of the drugs’ side effects and the amount of physical activity contributing to the serum levels of the tested biomarkers. One of the aims of the study was to evaluate short-term changes in physical function during the follow-up period. Since endurance and muscular strength are known to be reduced in LuTx recipients’ compared to healthy age- and gender-matched controls [9], physical function tests were not performed by the control group. We also lacked access to the BMD data of all patients, but BMD measurements and evaluations of bone microarchitecture at our transplant center have been reported previously [42].

Of the tested musculoskeletal markers, MSTN, SOST, Dkk 1, and PSTN differed between LuTx recipients and controls, thus suggesting the potential value of these markers in detecting the impairment of muscle function and bone function. Despite impressive improvements in the patients’ functional performance and lung function, the musculoskeletal markers did not significantly change over time. Nevertheless, long-term observations may reveal the normalization of these markers. In conjunction with clinical investigations and DXA scans, these markers may serve as useful surrogate measures for detecting musculoskeletal dysfunction in LuTx recipients. Since serum levels of MSTN were elevated in the short term as well as during follow-up in the long term, myostatin antibodies—which served as a promising treatment option for muscle-wasting disorders [43]—may be a treatment option for LuTx recipients worth to be investigated in the future.
